# A Plea for More Drastic Control

**Published:** 1921-05-21

**Authors:** 


### THE RECURRENCE OF RABIES.

### A Plea for More Drastic Control.

The occasional occurrence of cases of rabies among dogs since the outbreak in 1918 reminds us that the complete immunity from the disease we enjoyed here between 1902 and 1918 is, for the present, at an end. The advent of the aeroplane, together with the free movement of troops between the British Isles and France both by air and water, would seem to be responsible for the importation of the disease from abroad, at least during the war period, but it seems to us that since the peace increased vigilance is necessary both at our ports and aerodromes to prevent the smuggling of animals from abroad.

### Inadequate Punishment.

One feels that the present means of punishing dog smugglers by a fine is quite inadequate, since the class of person addicted to this breach of the law can easily afford a fine and is quite willing to take the risks. It was reported recently in our contemporary, the *Morning Post*, that

Lord Bledisloe contemplated introducing a Bill shortly to amend the Diseases of Animals Act, 1894, so that an offender against any existing order providing regulations for the importation of dogs shall be liable on conviction to six months' imprisonment, with or without hard labour, and we hope to see this or some similar legislation introduced at an early date.

THE PRESENT REGULATIONS.

Once a case of rabies has occurred our present methods are (1) to issue a muzzling order for the affected area, and (2) to restrict the movement of dogs from affected to non-affected areas, and (3) to destroy stray dogs. Now these methods are good as far as they go, but we would prefer the more drastic methods of muzzling all dogs in Great Britain for six to twelve months after a case has occurred—for only in this way will rabies be completely banished from the British Isles. The measures here advocated may seem too drastic, but we do not think they are more so than the occasion requires. A partial muzzling order only encourages the smuggling of dogs from affected to non-affected areas. Rabies is too serious a disease to be played with, and when there is a preventive measure in muzzling which, if properly applied, can eradicate the disease, why not use it?

